# Suicide in Victorian fathers

**DOI:** 10.1177/00048674251361660

**Published:** 2025-08-31

**Authors:** Alison Fogarty, Grace McMahon, Casey Hosking, Richard Fletcher, Jason Delgado, Dominique de Andrade, Liana Leach, Amanda Cooklin, Rebecca Giallo

**Affiliations:** 1 School of Psychology, Deakin University, Geelong, VIC, Australia; 2 Intergenerational Health, Murdoch Children’s Research Institute, Parkville, VIC, Australia; 3 Department of Paediatrics, The University of Melbourne, Parkville, VIC, Australia; 4 Fathers and Families Research Program, School of Health Sciences, The University of Newcastle, Callaghan, NSW, Australia; 5 Policy Department, Suicide Prevention Australia, Sydney, NSW, Australia; 6 Griffith Criminology Institute, Griffith University, Mt Gravatt, QLD, Australia; 7 School of Psychology, The University of Queensland, St. Lucia, QLD, Australia; 8 National Centre for Epidemiology and Population Health, The Australian National University, Canberra, ACT, Australia; 9 Judith Lumley Centre, La Trobe University, Bundoora, VIC, Australia

## Introduction

Suicide is a leading cause of death among Australian males aged 25–44 years (Australian Institute of Health and Welfare [AIHW], 2022), a time that often coincides with fatherhood. Despite this, fathers continue to be neglected in both perinatal and general mental health research and practice. Although it is well established that approximately one in 10 fathers experience depression during the postnatal period (Cameron et al., 2016; Giallo et al., 2023), far less is known about suicidal behaviours among fathers – both during the postnatal period and throughout fatherhood.

Our recent meta-analysis (Fogarty et al., 2024) identified only 14 studies which reported on the prevalence of suicidal ideation in fathers of young children (birth to 4 years). Although the pooled estimate of suicidal ideation was lower than expected (4.2%), we concluded that this was an underestimation due to major methodological limitations of the identified studies including (a) the exclusion of males with prior serious mental health problems and/or suicidal ideation and (b) the lack of suicide-specific measures. In addition, we identified over 200 articles which included mental health measures with items on suicidal ideation or behaviour but failed to report on this item separately, reflecting the neglect of this field of research.

### Study aims

Research relating to death by suicide in fathers is even more scarce, with no current evidence to our knowledge existing within Australia. To address this gap, the current study aimed to (1) determine the proportion of people dying by suicide in Victoria between 2017 and 2021 who were fathers of children ages 0–18 years, 0–4 years and 0–1 year and (2) describe the basic characteristics of these fathers who died by suicide.

## Methods

The research team reviewed multiple administrative data sets to determine the most feasible and accurate approach for identifying fathers of children aged 0–18 years who had died by suicide. National-level data sets, including the National Coronial Information System (NCIS), do not include linked birth records and therefore do not allow for the identification of fatherhood status. As a result, a state-based approach was adopted. This study analysed data from 2525 males who died by suicide in Victoria, Australia, between 1 January 2017 and 31 December 2021. We identified our cohort using data from the Victorian Death Index (VDI) and Victorian Death Index–Cause of Death (VDI-COD). Cause of death relating to suicide (coded as X60-X84) from the World Health Organization’s (WHO) International Statistical Classification of Disease and Related Health Problems (ICD-10) was sourced from VDI-COD, while demographic data were obtained from the VDI.

Data linkage through the Victorian Centre for Data Linkage was used to identify fatherhood status and corresponding child age at time of death. We obtained childbirth records from the Births database for births occurring between 1 January 1999 and 31 December 2021, where a male from our cohort was registered as the father. These data were linked with the Victorian death data to identify fathers of children aged 0–18 years who had died by suicide during our study period. This included confirmation of the linked Father Identification number and birth date of each child born during this period. Informed consent was not possible for this study. A waiver of consent and ethical approval was obtained from the Deakin University Human Research Ethics Committee (DUHREC 2024-078).

We calculated the mean age at the time of death, the father’s age at the birth of their first child and the age of the youngest child at their father’s death. Descriptive analysis was undertaken to determine the proportion of males who died by suicide who were fathers of a child/children aged: (a) 0–18 years, (b) 0–4 years and (c) 0–1 years. Social characteristics and suicide method were described for (a) the total sample, (b) non-fathers or those with a child >18 years and (c) fathers of a child 0–18 years.

## Results

Of the 2525 males who died by suicide in Victoria between 2017 and 2021, 24% (*n* = 607) were fathers with at least one child aged 0–18 years. Of these fathers, 7.7% (*n* = 47) were fathers of infants aged 0–1 years, and 32.8% (*n* = 199) were fathers of young children aged 0–4 years at the time of death.

Table 1 displays the key characteristics across groups. For fathers of children aged 0–18 years, the majority were Australian-born and partnered at the time of death. The mean age at death for males who were fathers was 42.4 years and the mean age at birth of the first child was 31 years. On average they had two children, with a mean age of the youngest child of 8.5 years. The most common method of suicide among fathers was hanging, strangulation or suffocation.

**Table 1.** Key characteristics (number and %) of men who died by suicide based on fatherhood status.

	Fathers of children 0–18 years (*N* = 607)	Not a father of children <18 years (*N* = 1918)	Total sample (*N* = 2525)
Country of birth (*n*, %)			
Australia	517 (85.2)	1414 (73.7)	1931 (76.5)
Born elsewhere	90 (14.8)	503 (26.2)	593 (23.5)
Not recorded	-	1 (< 0.1)	1 (< 0.1)
Relationship status (*n*, %)			
Partnered	363 (59.8)	600 (31.3)	963 (38.1)
Not partnered	232 (38.2)	1253 (65.3)	1485 (58.8)
Not recorded	12 (2.0)	65 (3.4)	77 (3.0)
Age at time of death in years (M, SD)	42.4 (9.3)	47.0 (19.8)	45.9 (18.0)
Range	21.5-76.8	10.1-96.6	10.1-96.6
Number of children (M, SD)	2.3 (1.3)	-	-
Age of youngest child at time of death (M, SD)	8.5 (5.5)	-	-
Range	0.1-18.9		
Method (*n*, %)			
Poisoning – drug	37 (6.1)	205 (10.7)	242 (9.6)
Poisoning – other	38 (6.3)	118 (6.2)	156 (6.2)
Hanging	418 (69.0)	1080 (56.3)	1498 (59.3)
Firearm or explosive material	41 (6.8)	135 (7.0)	176 (7.0)
Sharp or blunt object	12 (2.0)	64 (3.3)	76 (3.0)
Jumping from a height	10 (1.6)	101 (5.3)	111 (4.4)
Jumping or lying before a moving object	28 (4.6)	105 (5.5)	133 (5.3)
Intentional car crash	10 (1.6)	39 (2.0)	49 (1.9)
Other	13 (2.2)	71 (3.7)	84 (3.4)

## Discussion

This is the first study to examine suicide among fathers in Victoria, Australia, while parenting a child under 18 years. From 2017 to 2021, 607 fathers with children under 18 years old died by suicide in Victoria. This accounts for 24% of all male suicides in the state during this time and is equivalent to more than 120 fathers per year. During the critical postnatal period, 47 fathers of infants (<1 year) died by suicide, while 199 fathers of children under 4 years died during the early parenting period – nearly 40 per year. These findings highlight the need for targeted mental health support for fathers to encourage healthy coping strategies in the face of stress as well as address mental health symptoms where appropriate. Such support may be particularly important during early parenthood where stress often increases.

A small set of demographic information variables described our sample of males who died by suicide. Nearly 40% of fathers who died by suicide were not in a relationship at the time of death. This is almost twice the proportion of single-parent households with dependent children recorded in the 2021 Australian Census (Australian Institute of Family Studies [AIFS], 2023). Relationship difficulties and breakdowns are a well-established risk factor for suicide (Wilson et al., 2025), and our findings further highlight the need for father-inclusive support services that promote healthy communication and relationship stability. Evidence-based family or co-parenting interventions, such as Family Foundations (Feinberg et al., 2016), are needed which engage all parents/caregivers to manage the impact of stress on family relationships, promote parental mental health and reduce conflict. In addition, post-separation support is essential, particularly for parents of young children navigating custody arrangements and family court proceedings. Moreover, the mean age of children at the time of their fathers’ death was 8 years. While few studies have examined child outcomes following paternal suicide, losing a parent to suicide is a known risk factor for mental health problems and risk of suicide in adulthood (Guldin et al., 2015; Melhem et al., 2008). Thus, comprehensive bereavement and mental health support for children and families following paternal suicide is crucial in helping to mitigate long-term intergenerational impacts.

### Strengths and limitations

This study represents an important first step in assessing the feasibility of using administrative data to examine suicide among fathers. A key limitation is that only birth records from Victoria were accessible, meaning fathers of children born interstate or overseas may not have been identified. However, given that it was not possible to determine children’s ages through other data sets (e.g. suicide registries), we believe this method – while likely a conservative estimation – provides a practical and feasible approach for examining this issue. Moreover, the use of administrative data to describe the relationship status may not be accurate, as official records are not likely to reflect relationship separations that occurred shortly prior to death. We were also limited in the socio-economic variables available within the VDI data set, such as income, employment status or housing insecurity – all of which may be important contextual factors in understanding paternal suicide. Future studies should aim to link additional administrative data sets to capture these contextual factors.

## Conclusion

Suicide prevention is a key national priority, highlighted by Australia’s recent establishment of the National Suicide Prevention Office. While males account for 75% of all suicides (World Health Organization [WHO], 2014), paternal suicide has received limited attention. Although fatherhood is known to be a protective factor against suicide (Dehara et al., 2021), our findings suggest that suicide risk should not be neglected among fathers and highlight the importance of considering the complexities of fatherhood in prevention efforts. There is a critical need to understand the contextual and relational factors within fatherhood that may contribute to suicidal behaviour and death by suicide. This includes factors such as relationship problems, pre-existing mental health problems, court proceedings, police involvement and family violence, as well as their interactions with services leading up to their deaths. This evidence is crucial for developing early intervention responses to support fathers experiencing distress and prevent the long-term impact of losing a father to suicide on children and families.
